# Characteristics of retracted articles in ophthalmology

**DOI:** 10.1016/j.heliyon.2024.e35460

**Published:** 2024-07-30

**Authors:** Yosra Er-Reguyeg, Christophe Boudry, Frederic Mouriaux

**Affiliations:** a Faculté de Médecine. Université Laval, Québec, Canada; b Normandie Univ, UNICAEN, Média Normandie, Caen, France; c URFIST, Ecole Nationale des Chartes, PSL Research University, Paris, France; d Ophthalmology Department, CHU Rennes-Rennes University, France; e CUO-Recherche, Centre de Recherche du CHU de Québec – Université Laval, Axe Médecine Régénératrice, Hôpital du Saint-Sacrement, Québec, Canada

## Abstract

The retraction of publications is a crucial aspect of scientific integrity; it aims to correct the literature and alert scholars and the general public by identifying and labelling articles that contain erroneous data, unreliable findings, or flawed conclusions. Identifying and characterizing retracted articles within the scientific literature is thus very important. The aims of this article were to characterize retracted articles in the ophthalmological literature. One hundred and fifty-one retracted articles published between 1966 and 2023 were retrieved. The number of retracted articles showed an upward trend from 2020 onwards. Ocular oncology (n = 37, 24.5 %) was the most frequently represented subspeciality in the retracted articles, despite retina and uveitis being the most published. The most frequent reason for retraction was fake data (n = 62, 38 %). The labelling of retracted articles on some websites was unsatisfactory, especially on the free-access illegal platform Sci-Hub. On the other hand, platforms such as Dimensions, Scite and Retraction Watch exhibit promising accuracy. Improving the labelling of retractions is needed to reduce the citation of articles after they have been retracted. Solutions to reach this goal are discussed in this article.

## Highlights

• The number of retracted articles in ophthalmology showed an upward trend from 2020 onwards.
• Unexpectedly, retracted articles continue to be cited.
• The identification of retracted articles on most online databases is inadequate.

## 1 Introduction

Retraction is a crucial aspect of scientific integrity, it aims to correct the literature and alert scholars and the general public by labelling articles that contain erroneous data, unreliable findings, or flawed conclusions [1]. Although retractions are rare, with around five retractions per 10 000 published articles, numerous papers have highlighted an increasing proportion of retractions over time – in fact, the number has doubled in the last ten years – [[2], [3], [4]], and the field of medicine is no exception [5]. It is very difficult to know whether this increase is a result of a larger number of controversial papers because of an increase in fraud and errors [6] or whether the scientific community is improving its ability to detect and report these frauds and errors, thus reflecting its improved self-monitoring potential [[2], [3], [4],^7^,^8^]. Reasons for retraction can encompass a range of issues, such as scientific misconduct, errors, plagiarism, or ethical violations.

To avoid propagation of scientific errors, retracted articles must not be cited without referring to the retraction notices [9,10]. Unfortunately, it has been shown that retracted papers continue to be cited without reference to their retraction [9,11], as a result of inadequate labelling of retracted articles on publishers’ websites and in academic databases [[12], [13], [14]]. The spread of false information in the scientific literature through the retracted literature is a subject of great concern, as it could have significant effects on clinical practice and subsequent investigations [15,16]. It has also been shown that retracted articles entail significant cost and wastage of financial resources that could be put to better use [17].

In an effort to investigate and report on this matter, research investigating the retraction of articles has been published across several medical fields, including radiology, orthopaedics, and oncology, among others [[18], [19], [20]]. However, the data regarding retracted articles within the field of ophthalmology is not currently well defined, as very little literature has been published on the subject. Majumder et al. analysed the characteristics of 83 retracted ophthalmology articles identified on PubMed/Medline databases and published between 1994 and 2019 [21]. However, the fate of ophthalmology articles after their retraction has so far not been analysed. Therefore, the aims of this study were to identify and characterize retracted articles in the ophthalmology literature between 1966 and 2023, to analyse post-retraction citations, and to assess whether retracted articles were signalled as such on Web of Science (WoS), Google Scholar, and other platforms.

## 2 Materials and methods

A search for retracted papers to be included in this study was carried out on June 28, 2023, using the PubMed database (http://www.ncbi.nlm.nih.gov/pubmed), developed by the National Center for Biotechnology Information (NCBI) at the National Library of Medicine (NLM). PubMed was chosen because it is the most widely-used database in the field of medicine [22] and is considered by the ICMJE as the authoritative source for information about retractions [23]. Articles were extracted from PubMed using the following search strategy: "Retracted Publication" [Publication Type] AND journal article [Publication Type] AND “eye diseases” [MH] AND 1966:2023 [DP]“. “Journal article” includes the following publication types: journal articles, introductory journal articles, and reviews. The MeSH term “Eye Diseases” was chosen because it has been shown to have extensive coverage of the field of ophthalmology [24]. The year 1966 was selected as it corresponds to the initiation of the Medline database. No language restrictions were imposed. This publication search yielded a total of 151 articles. The retraction rate was obtained by dividing the number of retracted articles found by the number of articles related to eye diseases published each year. The retraction rates were normalized per 10000 published articles related to eye diseases. Data was processed and downloaded from PubMed as previously reported [24]. Reasons for retraction were assessed by consulting retraction notices on the publisher's website and were categorized, as presented in Table 1.

**Table 1:** Categories of reasons for article retraction. Table model design and Terminology inspired by [25].

General reason	Examples of specific reasons
Plagiarism	Overlap; duplication of text; manuscript plagiarized
Fake Data	Some data in the paper appeared identical; data integrity; numerous anomalies in the data; figures copied and manipulated; critical data in the paper was not valid
Duplicate Publication	Duplication of an article
Error/Mistake	Significant errors in the results; mistakenly republished data; errors in the data; one of the authors had mistakenly entered data in an incorrect location on a spread sheet; article was published before authors' corrections had been applied
Authorship Dispute	Resulting from disagreement between authors
Fake Review	The peer-review process provided false information
Copyright Issues	Infringement of copyright; other copyright issues
Others	Retractions did not come under the above reasons
Unknown	The reason for retraction is unclear, e.g. a paper retracted by the Editor and the Publisher; the paper contains substantial flaws in its scientific methodology

The numbers of article citations were retrieved on the Web of Science Core Collection database (Clarivate Analytics). Post-retraction citations were counted starting from one year after the date of retraction. The presence of retraction labelling (e.g. modified title with the mention “retracted”) was verified for the 151 references on the websites included in this study. Digital Object Identifiers (DOI) or titles of articles when a DOI was unavailable were used as queries. Full texts were searched and downloaded (if available) to ensure positive identification and to confirm accessibility by the public. The presence of any information (watermark, modified title with the mention of the term “retracted” …) enabling retraction identification was then checked in full texts (if available). All searches were conducted from July to August 2023. The presence of the 151 retracted articles studied was also checked on Retraction Watch Database, which is a specific platform serving to track and identify retracted articles.

The websites hosting references and/or full-text articles included in this study are presented in Table 2.

**Table 2:** Websites hosting references and/or full-text articles included in this study

Name of the platform	Type of website	Description or remarks
Publishers' websites		Full texts of paywalled articles on publishers' websites were searched using Laval University institutional access
The Web of Science Core Collection	Subscription-based database	Provided by Clarivate Analytics (http://www.webofknowledge.com/)
Google Scholar	Free bibliographic database	Freely accessible, with an automated approach, indexing any seemingly academic document that its crawlers can find and access on the web, including those behind paywalls, through agreement with their publishers. (https://scholar.google.com)
Researchgate	Academic social network	Provides a platform for academic users to share publications. (https://www.researchgate.net/)
Dimensions	Free bibliographic database	Inter-linked research information system provided by Digital Science (https://www.dimensions.ai). Freely accessible, Dimensions database was chosen among the newer academic databases because it is one of the most serious contenders of the Web of Science database [26]. (https://app.dimensions.ai/discover/publication)
Scite	Free bibliographic database	A platform for discovering and evaluating scientific articles via citations. This website also checks for retractions on Crossref or PubMed and uses its own algorithms to detect retracted articles. (https://scite.ai/home)
Unpaywall	Free service to locate open-access articles	Locates open-access articles and presents paywalled papers that have been legally archived and are freely available to users on other websites. (https://unpaywall.org/)
Open Access Button	Free service to locate open-access articles	Aggregates sources from repositories and opens access to journals. (https://openaccessbutton.org)
Sci-Hub	Illegal service	Provides free-of-charge access to the academic literature, despite the continued presence of paywalls. It is considered as black (illegal) open access. Does not ask for consent from authors or publishers, and raises many legal and ethical questions [27]. Searches using Sci-hub were performed from France, from a non-university Internet access. The authors' universities or institutions were therefore not involved in the downloading of articles via Sci-Hub.

SPSS Statistics Version 26 (IBM, Armonk, NY, USA) was used to conduct the statistical analysis. The normality of continuous variables was tested with Q-Q plots and Shapiro-Wilk's tests. Means and standard errors (SE) were used to present normally distributed continuous variables, medians and quartiles [first quartile (Q1) – third quartile (Q3)] for non-normally distributed continuous variables, and percentages for categorical variables. Pearson's Chi-Square test was used to assess significance for proportions between independent categorical variables. The Mann-Whitney-U test was used to assess significant differences in medians between two independent samples. The Wilcoxon signed-rank test was used to assess significant differences in paired samples not following a normal distribution. Statistical significance was set at a value of p < 0.05.

## 3 Results

### 3.1 General characteristics and trends over time for retracted literature

**Fig. 1:**
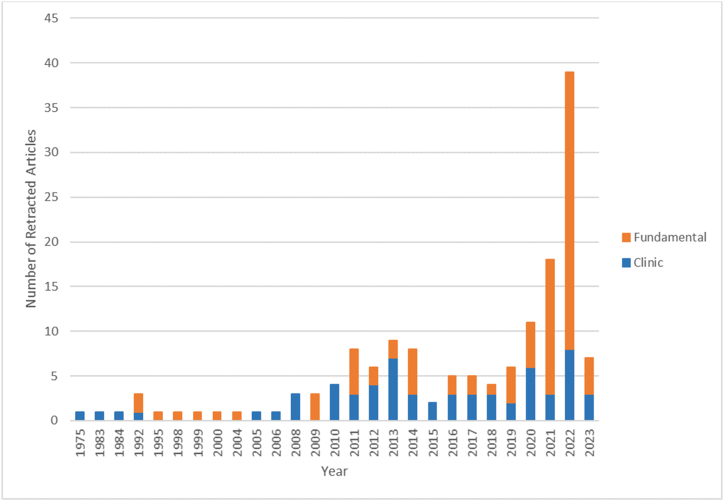
Number of articles retracted per year according to study type (2023 incomplete).

One hundred and fifty-one retracted articles were retrieved and included in this study. The overall retraction rate was 2.77/10 000 publications over the studied period (151 articles related to eye diseases were retracted among 544472 articles published during the same period). As shown in Fig. 1, the number of retracted articles has increased significantly since 2020. The 3 years with the most retractions were, in descending order, 2022 (n = 39, 25.8 %), 2021 (n = 18, 11.9 %), and 2020 (n = 11, 7.3 %) and these three years accounted for 45 % (n = 68) of all retractions. Fundamental science studies accounted for 55 % (n = 88) of retracted articles, while 45 % (n = 68) were clinical studies. Interestingly, the proportion of retracted fundamental science studies rose in the past three years (2021–2023). The field of ophthalmology encompasses various subspecialties that specifically address particular eye diseases or specific parts of the eye. Studies from a variety of ophthalmic subspecialties were retracted. Table 3 summarizes the subspecialties covered by the 151 articles. Although the most prevalent eye diseases are age-related macular degeneration, cataract, diabetic retinopathy, and glaucoma [28], and although the largest numbers of published articles in ophthalmology relate to uveitis, retina and glaucoma [[29], [30], [31]], we found that ocular oncology was the most common ophthalmic subspeciality in retracted articles."

**Table 3:** Subspecialties covered in 151 retracted articles in the field of ophthalmology.

Ophthalmic Subspeciality	Specific eye structures/conditions targeted	Examples of conditions included	Number of Articles Retracted
			No. (%)
Ocular Oncology	Ocular tumours	Retinoblastoma, primary/metastatic ocular malignancies, adnexal ocular tumours	37 (24.5)
Posterior Segment	Retina, vitreous, choroid	Age-related macular degeneration, retinal detachment	37 (24.5)
Ocular Surface	Cornea, conjunctiva	Keratitis, conjunctivitis, episcleritis	17 (11.3)
Anterior Segment	Iris, lens, anterior chamber, trabecular meshwork	Cataracts, glaucoma	16 (10.6)
Paediatrics	Conditions specific to children	Amblyopia, paediatric strabismus	12 (7.9)
Oculoplastics	Eyelids, lacrimal conducts, orbit	Grave's orbitopathy, orbital fractures, eyelid malpositioning	9 (6.0)
Neuro-ophthalmology	Optic nerve, optic pathway	Optic neuropathy, papilledema, adult strabismus	8 (5.3)
Ocular inflammation/immunology	Uvea, inflammatory conditions	Uveitis, endophthalmitis, scleritis	8 (5.3)
Refractive	Refractive error correction	Myopia, hyperopia, astigmatism, presbyopia	4 (2.6)
Other			3 (2.0)

**Fig. 2:**
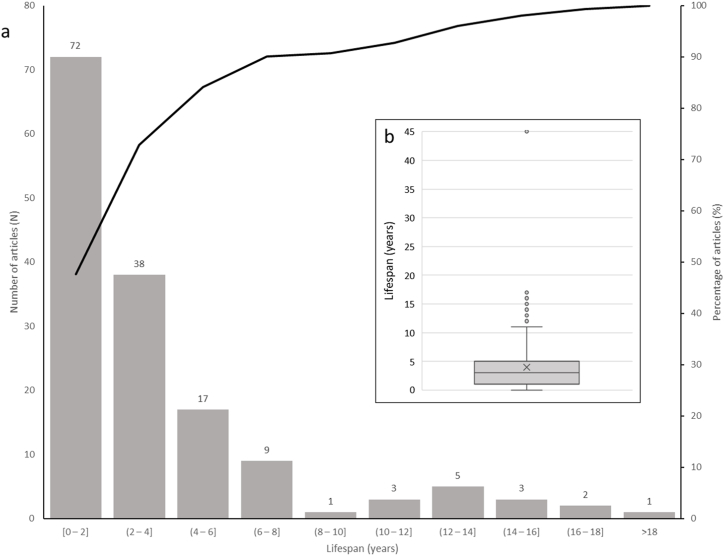
Lifespan of articles.

The lifespan of the articles, which corresponds to the period of time between publication and retraction [32], is presented in Fig. 2. Ninety per cent (n = 136) of all articles were retracted within 8 years of their publication and 47.7 % (n = 72) were retracted within 2 years of publication. The article with the longest lifespan was retracted 45 years after publication.

### 3.2 Reasons for retraction

The reasons for retractions are numerous, including scientific misconduct, errors, plagiarism, or ethical violations. In all, 163 retraction reasons were recorded. One hundred and thirty-three articles had one reason for retraction, while 18 had two or more. Fake data (n = 62, 38.0 %) and errors/mistakes (n = 37, 22.7 %) were the most frequent reasons of retraction (Table 4).

**Table 4:** Frequencies of reasons of retraction.

Reason of Retraction	Frequency of retraction reason
	No. (%)
Fake Data	62 (38.0)
Error/Mistake	37 (22.7)
Authorship Dispute	14 (8.6)
Others	14 (8.6)
Plagiarism	12 (7.4)
Duplicate Publication	11 (6.7)
Unknown	12 (7.4)
Copyright Issues	1 (0.6)
Sum of reasons for retraction	163 (100.0)

### 3.3 Journal and author characteristics

The 151 articles analysed were published across 91 journals. Table 5 shows that no single journal had substantially more retracted articles, as the top 10 journals with the largest numbers of retractions accounted for only 31.1 % of retractions. However, if we divide the number of retracted articles by the total number of articles published under the MeSH term “eye disease”, some journals had a noticeably larger proportion of retracted articles (10.8 %), such as AAPS (in the case of PharmSciTech) and Oncology reports (9.8 %). As for the ophthalmic subspecialities (Table 3), oncology was predominant.

**Table 5:** Top 10 Journals with the most retractions out of the 151 retracted articles in the field of ophthalmology. Searching PubMed to obtain the number of ophthalmology articles published per journal used the terms ("Journal title"[Journal]) AND (eye diseases [MeSH Terms]).

Journal	Number of retracted articles	Number of retracted articles/Total number of articles published in the field of ophthalmology
	No. (%)	Fraction (%)
PLoS One	8 (5.3)	8/4314 (0.185)
Current Eye Research	6 (4)	6/3583 (0.167)
Oncology Reports	5 (3.3)	5/51 (9.803)
Biomedicine & Pharmacotherapy	4 (2.6)	4/173 (2.312)
Molecular Vision	4 (2.6)	4/2422 (0.165)
British Journal of Ophthalmology	4 (2.6)	4/14 785 (0.027)
Oxidative Medicine and Cellular Longevity	4 (2.6)	4/139 (2.878)
AAPS PharmSciTech	4 (2.6)	4/37 (10.811)
Bioscience Reports	4 (2.6)	4/89 (4.494)
Graefe's Archive for Clinical and Experimental Ophthalmology	4 (2.6)	4/6955 (0.058)
Total	47 (31.1 %)	

**Fig. 3:**
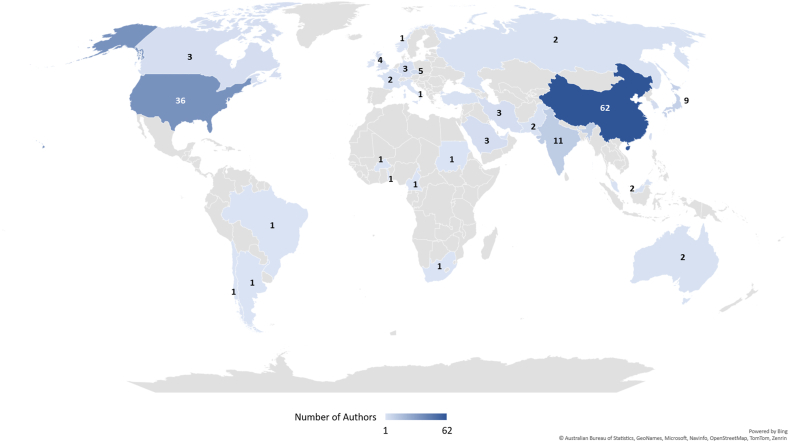
Number of authors with retracted articles per country.

An interesting aspect to explore is that of the level of retractions per country. The world map presented in Fig. 3 illustrates the numbers of authors concerned for each country. In all, 172 authors were found. It can be remarked that 7 articles were written by the same Japanese author, Yoshitaka Fujii. All of them were randomized controlled trials related to the use of various anaesthetic agents in paediatric strabismus surgery. This author had the second largest number of scientific paper retractions (172 articles) for a single author as of September 2023 [33]. Three other authors were recurrent offenders, with two articles retracted for each. For these three authors, the two articles had similar titles, taking the form of either follow-up studies or research in the same field, and the reasons for retraction were similar, entailing fake data or errors/mistakes in their methodology or results.

### 3.4 Citations

Our study also analysed post-retraction citations. In order to eliminate cases of citation by authors potentially unaware that the cited article had been retracted, we analysed post-retraction citations starting from one year after retraction. The median numbers of citations pre-retraction and post-retraction were 4.0 [1.0–16] and 1.0 [0.0–5.0], respectively, and the median total number of citations was 12.0 [4.8–26.3]. We found that the number of citations significantly decreased after retraction (p < 0.001).

Further to this, we determined whether the articles were less often cited after retraction when information about retraction status was included in the title of the article. To do this, we compared the median number of post-retraction citations between articles that included the word "retraction" in the title and those that did not. We did not find any statistically significant difference between the median number of post-retraction citations of articles with information about retraction in the title and the median number for those without information about retraction in the title (1.00 [0.00–3.00] vs. 1.00 [0.00–6.25]; p = 0.217).

### 3.5 The labelling of retracted articles on publishers’ websites and various databases

**Fig. 4:**
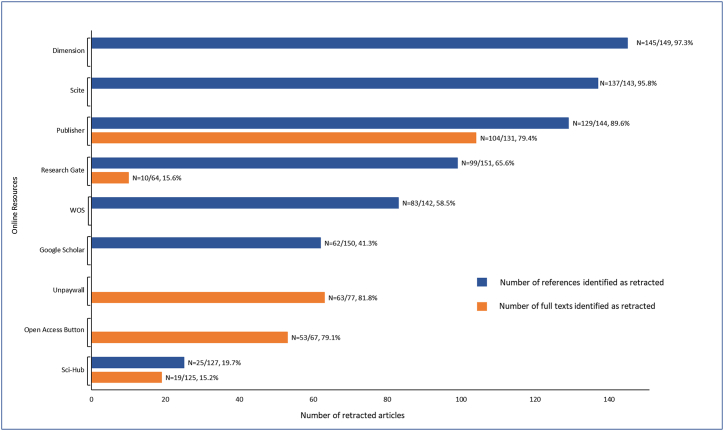
Labelling of retraction across publishers' websites and various databases. The first number of each fraction indicates the number of references or full texts identified as retracted on each site. The second number indicates the number of references or full texts retrieved (identified and unidentified as retracted) on each platform; the total number of articles studied was 151.

In order to avoid the diffusion of scientific errors through citations of retracted articles (references and full texts) these articles should be labelled as retracted in databases. Indeed, in order to be considered, retractions should be identifiable and visible to researchers, e.g. by adding the term “retracted” to the title of the references of articles or by watermarking each page of full text diagonally as “retracted” or “withdrawn”. Fig. 4 depicts the number of retracted articles identified as retracted across all analysed platforms. It is important to note that more than 10 % of references and more than 20 % of full texts are not identified as retracted on publishers’ websites, evidencing that some of them do not follow the COPE guidelines on retraction [1]. Notably, Dimension and Scite stood out as the two most effective platforms in accurately identifying retractions among referenced articles (97.3 % and 95.8 %, respectively). In contrast, open access platforms (Unpaywall and Open Access Button) identified about 80 % of full texts as retracted, which is far from satisfactory.

We also sought to assess whether the indication of retraction in the title of the article in the references on the publishers' websites influences the labelling of retractions on other sites hosting the references. Indeed, information on article retraction should in principle appear first on the publishers' websites and should then be disseminated to other research platforms and servers. Seventy-four articles (49 %) had their retraction directly indicated in the title on the publisher's website, 71 (47 %) did not have their retraction directly indicated in the title, and 6 (4 %) were not available because the articles had been removed from the publisher's website. Interestingly, retraction indications in the title on publishers' websites significantly increased the number of articles labelled as retracted on Google Scholar and on the WoS (p < 0.001 and p = 0.036 respectively). Conversely, it did not significantly increase the number of articles identified as retracted on other platforms. This result shows that the indication of retraction in the title of an article in the references of publishers' websites has an impact on the labelling of retractions on other sites hosting references.

## 4 Discussion

The continuing use of retracted literature has been described for over 30 years [34] and is extensively documented [9,35,36]. The present analysis of retracted articles in the field of ophthalmology provides valuable insights into the landscape of scientific research and publication. Nevertheless, it has some limitations. The present analysis was limited to the articles available in PubMed, so that is could have underestimated the overall number of retractions. The retrospective nature of this bibliographic study, encompassing 151 retracted articles from 1966 to 2023, may have introduced bias related to evolving publishing practices and technological advancements over the years. Furthermore, the reliance on specific platforms for data retrieval could have resulted in overlooking articles not included in these sources. Nevertheless, reporting on the characteristics of retracted publications is crucial. It alerts the scientific community to issues in scientific publishing, mobilizes publishers and authors to prevent such problems, and helps clinicians to avoid the propagation of errors in the scientific literature. In addition, the substantial impact of retractions affects various dimensions, ranging from financial implications as a result of wasted resources [17], to spurious clinical decision-making potentially affecting patient care [15].

The number of retracted articles in ophthalmology has increased significantly in recent years, as with other medical specialities [37]. Interestingly, a significant proportion (n = 72, 47.7 %) of the articles studied here were retracted within just 2 years of publication, suggesting that issues had been identified relatively soon after publication. The main reason for retraction was fake data (38 %), followed by error/mistake (22.7 %). A subset of articles involved several reasons for retraction, highlighting complex scenarios leading to their withdrawal. Earlier studies have pointed out that it is difficult to know whether the global increase in retracted articles is a result of a larger number of controversial papers because of a rise in fraud and errors [6] or whether the scientific community is improving its ability to detect and report such fraud and errors, thus reflecting its enhanced self-monitoring abilities. Nevertheless, articles retracted for fake data or error/mistakes are also certainly a result of the appearance of platforms such as PubPeer in 2012, which enables users to make comments on scientific articles post-publication, and to report suspicions of breaches of scientific ethics, which can be the cause of retractions. No article published before 2006 was retracted for plagiarism/duplication, suggesting the scientific ecosystem's ability to address these concerns with time.

As observed, retracted articles continue to be cited, despite what might be expected. However, our data demonstrates a significant reduction in the number of citations following retraction. Continuing citation of retracted articles mainly stems from inadequate identification (references and PDF) on hosting sites [14,38]. Our data indicates that ophthalmology is no exception. We demonstrated that the identification of retracted articles on some platforms hosting articles was far from ideal, particularly with Google Scholar and the unauthorized free-access website Sci-Hub, which both lag behind on updating retracted literature. This could be attributed to the fact that article references on these platforms mostly consist of the title of the article in the case of Google Scholar (title + preview of abstract), and only consist of the title of the article for Sci-Hub. More recent platforms such as Dimensions and Scite, on the other hand, exhibit greater accuracy. It would be interesting to conduct studies in the future to assess whether the least efficient platforms have implemented procedures to better identify retracted articles, and whether they are more efficient.

There are solutions to limit the citation of retracted articles and to avoid the propagation of scientific errors [1,14,[38], [39], [40]]. As previously suggested [38,41], a potential solution could involve the consistent incorporation of retraction information in the titles of retracted articles on the publisher's websites, using the term "retracted" at the beginning of the title. Our findings indicate that this recommendation is still far from being met, as only 49 % (n = 74) of articles had the retraction directly specified in the title of the reference on publishers' websites. However, the presence of retraction information directly in the article title did not significantly impact the post-retraction citation rates, although they were higher when retraction was not indicated in the title. This suggests that applying this recommendation alone will not necessarily solve the problem. Nevertheless, retraction indications in the title on publishers' websites significantly increased the number of articles identified on Google Scholar and on the WoS. Conversely, it did not significantly increase the number of articles identified on other websites, especially on Dimension and Scite. This could be explained by the fact that these websites do not consider information provided in the title to identify retractions, but rather use other methods.

Clearly, researchers play a capital role in preventing citations of retracted articles without acknowledging their retraction. Tools such as Zotero or EndNote reference management software can be helpful in identifying retracted articles effectively. They have built-in systems for alerting researchers when a retracted article is stored in their article database, via checks on the Retraction Watch database. In the present study, using Zotero or EndNotes would have identified 100 % of the retracted articles studied, regardless of whether they were correctly identified on the website used to retrieve them. Publishers have the opportunity to reduce the number of articles citing retracted content, by asking authors to confirm the exclusion of such articles and by helping them to identify them in their references. Unfortunately, at present, only a small number of journals have adopted this practice.

In conclusion, our study provides extensive insight into retracted literature in the field of ophthalmology. It characterizes the retracted literature in detail, and analysed post-retraction citation, which had never been done before in the speciality. It also highlights solutions that could be implemented simply to limit the citation of retracted articles, and thus limit the propagation of scientific errors in the ophthalmologic literature.

## Data availability statement

Data will be made available on request.

## CRediT authorship contribution statement

**Yosra Er-Reguyeg:** Writing – review & editing, Writing – original draft, Visualization, Validation, Methodology, Investigation, Formal analysis, Data curation. **Christophe Boudry:** Writing – review & editing, Writing – original draft, Visualization, Validation, Supervision, Resources, Project administration, Methodology, Investigation, Formal analysis, Data curation, Conceptualization. **Frederic Mouriaux:** Writing – review & editing, Writing – original draft, Visualization, Validation, Supervision, Resources, Project administration, Methodology, Investigation, Formal analysis, Data curation, Conceptualization.

## Declaration of competing interest

The authors declare that they have no known competing financial interests or personal relationships that could have appeared to influence the work reported in this paper.
